# Accuracy of glomerular filtration rate estimation equations in patients with hematopathy

**DOI:** 10.7555/JBR.32.20160172

**Published:** 2017-07-30

**Authors:** Han Sun, Xiaohua Pei, Weihong Zhao

**Affiliations:** 1. Division of Nephrology; 2. Division of Respiratory Medicine, Department of Geriatrics, The First Affiliated Hospital of Nanjing Medical University, Nanjing, Jiangsu 210029, China.

**Keywords:** creatinine, cystatin C, equation, glomerular filtration rate, hematopathy

## Abstract

Renal dysfunction is a common side-effect of chemotherapeutic agents in patients with hematopathy. Although broadly used, glomerular filtration rate (GFR) estimation equations were not fully validated in this specific population. Thus, this study was designed to further assess the accuracy of various GFR equations, including the newly 2012 CKD-EPI equations. Referring to ^99m^Tc-DTPA clearance method, three Scr-based (MDRD, Peking, and CKD-EPI_Scr_), three Scys C-based (Steven 1, Steven 2, and CKD-EPI_Scys C_), and three Scr-Scys C combination based (Ma, Steven 3, and CKD-EPI_Scr-Scys C_) equations were included. Bias, P_30_, and misclassification rate were applied to compare the applicability of the selected equations. A total of 180 Chinese hematological patients were enrolled. Mean bias, absolute mean bias, P_30,_ misclassification rate and Bland-Altman plots of the CKD-EPI_Scr-Scys C_ equation were 7.90 mL/minute/1.73 m^2^, 17.77 mL/minute/1.73 m^2^, 73.3%, 38% and 79.7 mL/minute/1.73 m^2^, respectively. CKD-EPI_Scr-Scys C_ predicted the most precise eGFR both in lymphoma and leukemia subgroups. Additionally, CKD-EPI_Scys C_ equation in the rGFR ≧ 90 mL/minute/1.73 m^2^ subgroup and Steven 2 equation in the rGFR<90 mL/minute/1.73 m^2^ subgroup provided more accurate estimates in each subgroup. The CKD-EPI_Scr-Scys C_ equation could be recommended to monitor kidney function in hematopathy patients. The accuracy of GFR equations may be closely related with GFR level and kidney function markers, but not the primary cause of hematopathy.

## Introduction

Chronic kidney disease (CKD) has been a major health problem worldwide. Moreover, the incidence of CKD has been sharply expanding^[1–2]^. A cross-section survey in China demonstrated the prevalence of CKD reached 10.8%, equivalent to 119.5 million CKD subjects^[3]^. The incidence of renal impairment in patients of hematopathy has been increasing^[4]^. Acute renal impairment is commonly associated with early treatment-related toxicities that lead to severe hemodynamic disturbances, most notably hepatic veno-occlusive disease (VOD) and sepsis, and with the use of nephrotoxic medication^[5–6]^. Chronic renal impairment is commonly attributed to delayed effects of the infiltration of kidneys by leukemic cells, nephrotoxicity, and metabolic changes arising from chemotherapy, radiotherapy, infections, and intravascular coagulo-pathy^[7–11]^. A recent study has indicated that 20%-50% of multiple myeloma patients required dialysis after 15 years of illness^[12]^. Kidney Disease Improving Global Outcomes (KDIGO) in 2012 proposed that hematopathy-associated renal impairment should be regarded as a special kind of CKD^[13]^, requiring regular monitoring of urine, blood pressure and GFR^[14–16]^.

As the best overall measurement of kidney function, the determination of GFR has three kinds. One is inulin clearance, which is regarded as the gold standard. Whereas, this impractical standard measurement of GFR is cumbersome, costly, and therefore not commonly available^[17]^. The second method is isotope plasma clearance, a substitution for inulin clearance, slightly simpler than the former in operation procedures, but also as accurate as the former. However, the isotope plasma clearance is also costly, and radioactive, just available in scientific research. The third kind is GFR evaluation equations, which now have been recommended to assess kidney function as a conventional method^[18]^.

The GFR evaluation equations were first constructed in 1976 by Cockcroft-Gault. After several generations were developed, the equations have experienced serum creatinine (Scr) based equations, serum cystatin C (Scys C) based equations and Scr-Scys C combination based equations. Several hundreds of equations were developed and validated in various ethnicities and CKD. However, few researchers focused on the subjects with hematopathy-associated renal impairment, who, more than ever, need accurate, noninvasive and repeatable methods to monitor kidney function. By far, no studies paid attention to this special population. Thus, this study was designed to validate whether the 2012 CKD-EPI equations were also accurate or not in hematological subjects, in comparison with other GFR equations ( ***Table 1***).

**Tab.1:** Equations to predict glomerular filtration rate

	**Scr**	**Scys C**	**Gender**	**Equation**	**Years**	**Subjects **	**Disease**	**Race**
**Scr-based**								
MDRD				186×Scr^-1.154^×Age^-0.203^×( 0.742, if female)	1999	1,628	CKD	American
Peking				175×Scr^-1.234^×Age^-0.179^×( 0.79, if female)	2006	1,570	CKD	Chinese
CKD-EPI_ Sc__r_	≤0.7		Female	144×(Scr/0.7)^−^^0.329^×(0.993)^Age^×(1.159 )	2009	12,150	CKD	American
	>0.7			144×(Scr/0.7)^−^^1.209^×(0.993)^Age^×(1.159 )				
	≤0.9		Male	141×(Scr/0.9)^−^^0.411^×(0.993)^Age^×(1.159 )				
	>0.9			141×(Scr/0.9)^−^^1.209^×(0.993)^Age^×(1.159 )				
**Scys** C**-based**								
Steven 1				76.7×Scys C^−^^1.19^	2008	3,418	CKD	American
Steven 2				127.7×Scys C^−^^1.17^×Age^−^^0.13^×(0.91 if female)	2008	3,418	CKD	American
CKD-EPI_Scys C_		≤0.8		133×(Scys C/0.8)^−^^0.499^×0.996^Age^×(0.932 if female)	2012	12,150	CKD	American
		>0.8		133×(Scys C/0.8)^−^^1.328^×0.996^Age^×( 0.932 if female)				
**Scr and Scys C-based**								
Ma				169×Scr^-^^0.608^×Scys C^-^^0.63^×Age^-^^0.157^×(0.83 if female)	2007	684	CKD	Chinese
Steven 3				177.6×Scr^−^^0.65^×Scys C^−^^0.57^×Age^−^^0.20^×(0.82 if female)	2008	3,418	CKD	American
CKD-EPI_Sc__r__-Scys C_	≤0.7	≤0.8	Female	130×(Scr/0.7)^−^^0.248^×(Scys C/0.8)^−^^0.375^×0.995^Age^	2012	12,150	CKD	American
		>0.8		130×(Scr/0.7)^−^^0.248^×(Scys C/0.8)^−^^0.711^× 0.995^Age^				
	>0.7	≤0.8		130×(Scr/0.7)^−^^0.601^×(Scys C/0.8)^−^^0.375^× 0.995^Age^				
		>0.8		130×(Scr/0.7)^−^^0.601^×(Scys C/0.8)^−^^0.711^× 0.995^Age^				
	≤0.9	≤0.8	Male	135×(Scr/0.9)^−^^0.207^×(Scys C/0.8)^−^^0.375^× 0.995^Age^				
		>0.8		135×(Scr/0.9)^−^^0.207^×(Scys C/0.8)^−^^0.711^×0.995^Age^				
	>0.9	≤0.8		135×(Scr/0.9)^−^^0.601^×(Scys C/0.8)^−^^0.375^×0.995^Age^				
		>0.8		135×(Scr/0.9)^−^^0.601^×(Scys C/0.8)^−^^0.711^×0.995^Age^				

Scr: serum creatinine, shown as mg/dL; Scys C: serum cystatin C, shown as mg/L.

## Subjects and methods

### Subjects

A total of 180 Chinese participants with hematopathy, who were outpatients or inpatients of the First Affiliated Hospital of Nanjing Medical University between December 2009 and December 2015, were enrolled in the study. All participants provided their written informed consent. The study was conducted in accordance with the Declaration of Helsinki and approved by the ethics committee of the First Affiliated Hospital of Nanjing Medical University.

Subjects with acute kidney injury, severe edema, skeletal musclepleural effusion or ascites, malnutrition, amputation, heart failure or ketoacidosis were excluded. Additionally, subjects who were taking glucocorticosteroids, renal replacement therapy were also excluded. The subjects were divided into two subgroups, the lymphoma group and the leukemia group. Therefore, the GFR equations were compared not noly in the reference GFR (rGFR) levels (rGFR≥90 and<90 mL/minute/1.73 m^2^), but also in this two subgroups.

### Determination of Scr and Scys C

Scr concentration was assayed by isotope dilution mass spectrometry (IDMS) traceable standardized enzymatic method (Kehua Dongling Diagnostic Products Co., Ltd., Shanghai, China), with a reported coefficient of variation of 6%, reference range 44-136 mmol/L. Scys C was examined by the particle-enhanced immunoturbidimetry method (Leadman Biomedical Co., Ltd., Beijing, China), with a reported coefficient of variation of 8%, reference range 0.60-1.55 mg/L. Both fasting serum samples were assayed on an Olympus AU5400 autoanalyser (Olympus Co., Japan).

### Measurement and estimation of GFR

rGFR was measured using ^99m^Tc-diethylene triamine pentaacetic acid (^99m^Tc-DTPA) kidney dynamic imaging^[19]^ on a single photon emission computed tomography (Siemens Co., Germany). Participants received a bolus injection in the elbow vein of 185 MBq ^99m^Tc-DTPA (Nanjing Senke Co., China, purity 95%–99%), after oral hydration with 300 mL water, and then emptying the bladder. rGFR was automatically calculated on the computer with the Gates method after image acquisition^[20]^.

eGFR was calculated separately from GFR estimation equations, including Modification of Diet in Renal Disease (MDRD)^[21]^, Peking^[22]^, Steven 1 based on Scys C^[23]^, Steven 2 based on Scys C^[23]^, Steven 3 based on Scr and Scys C^[23]^, Ma based on Scr and Scys C^[22]^, Chronic Kidney Disease Epidemiology Collaboration (CKD-EPI) equation based on Scr (CKD-EPI _Scr_)^[24]^, CKD-EPI equation based on Scys C (CKD-EPI _Cys C_)^[25]^, and CKD-EPI equation based on Scr and Scys C (CKD-EPI _Scr-Scys C_)^[25]^.

### Statistical analyses

Bias, precision, and accuracy were calculated to compare the performance of the equations. Bias was defined as the mean difference between eGFR and rGFR (eGFR-rGFR). Absolute bias was equal to the absolute mean difference |(eGFR-rGFR)|. Precision was expressed as inter-quartile range (IQR) (25%–75%). P_30_ was determined as the proportion of eGFR within± 30% of rGFR.

Additionally, Bland-Altman analysis^[26]^ was also calculated to compare the 95% limits of agreement (LOA, mean Bias±1.96 SD) of the equations. The smaller the LOA, the greater precision. Wilcoxon matched-pairs signed rank test was used to compare the bias, and the McNemar test was used to compare P_30_. *P*<0.05 was considered as statistically significant. The calculation and statistical analysis above were performed with SPSS software (version 20.0; SPSS, Chicago, IL, USA) and Medcalc (ver. 15.2 for Windows; MedCalc Software, Mariekerke, Belgium).

## Results

### General clinical characteristics

A total of 180 Chinese participants with hematopathy in the First Affiliated Hospital of Nanjing Medical University between December 2009 and December 2015 were enrolled in this study. Their mean age was 40.56±13.95 years. The mean level of Scr, Scys C and rGFR were 0.78 mg/dL, 1.09 mg/L and 87.54 mL/minute/1.73 m^2^, respectively. The mean values for the eGFRs varied from 80.42 mL/minute/1.73 m^2^ to 139.57 mL/minute/1.73 m^2^. The rGFR<90 mL/minute/1.73 m^2^ group consisted of 96 subjects. The rGFR≧90 mL/minute/1.73 m^2^ group was composed of 84 subjects. The detailed laboratory and anthropometric measurements are shown in ***Table 2***.

**Tab.2:** Demographic and clinical characteristics

**Subjects**	**Total**	**rGFR<90 mL/minute/1.73 m**^2^	**rGFR≥≧90 m**L**/minute/1.73 m**^2^
Number (male/female)	180(103/77)	96(63/33)	84(40/44)^*^
Age, years	40.56±13.95	44.80±13.28	35.71±13.17
Height, cm	166.20±6.66	167.58±6.03	165.05±6.89^**^
Weight, kg	62.37±8.25	64.66±7.36	61.04±8.91^**^
BMI, kg/m^2^	22.53±2.15	22.74±2.13	22.05±2.32^**^
**Renal variables**			
Scys C, mg/l	1.09±0.49	1.28±0.59	1.01±0.11^**^
Scr, mg/dl	0.78±0.48	1.08±0.56	0.69±0.47^**^
Albumin, g/l	41.10±4.5	41.25±4.88	41.06±4.47
rGFR, mL/minute/1.73m^2^	87.54±21.05	71.98±13.70	105.42±11.77^*^
**Type**s** of hematopathy**			
Lymphoma	88(48.9)	52(59.1)	36(40.1)
Leukemia	63(35.0)	29(46.0)	34(54.0)
Multiple myeloma	20(11.1)	10(50.0)	10(50.0)
Anemia	6(3.3)	4(66.7)	2(33.3)
Myelodysplastic syndrome	3(1.7)	1(33.3)	2(66.7)

Cell values represent mean (SD) and N (%). Scr: serum creatinine; Scys C: serum cystatin C; rGFR: reference glomerular filtration rate; eGFR: estimated glomerular filtration rate. ^*^ *P *<0.05, ^**^ *P*<0.001, compared with the rGFR<90 mL/minute/1.73 m^2^ group.

### Accuracy of the equations in the whole population

**Fig.1:**
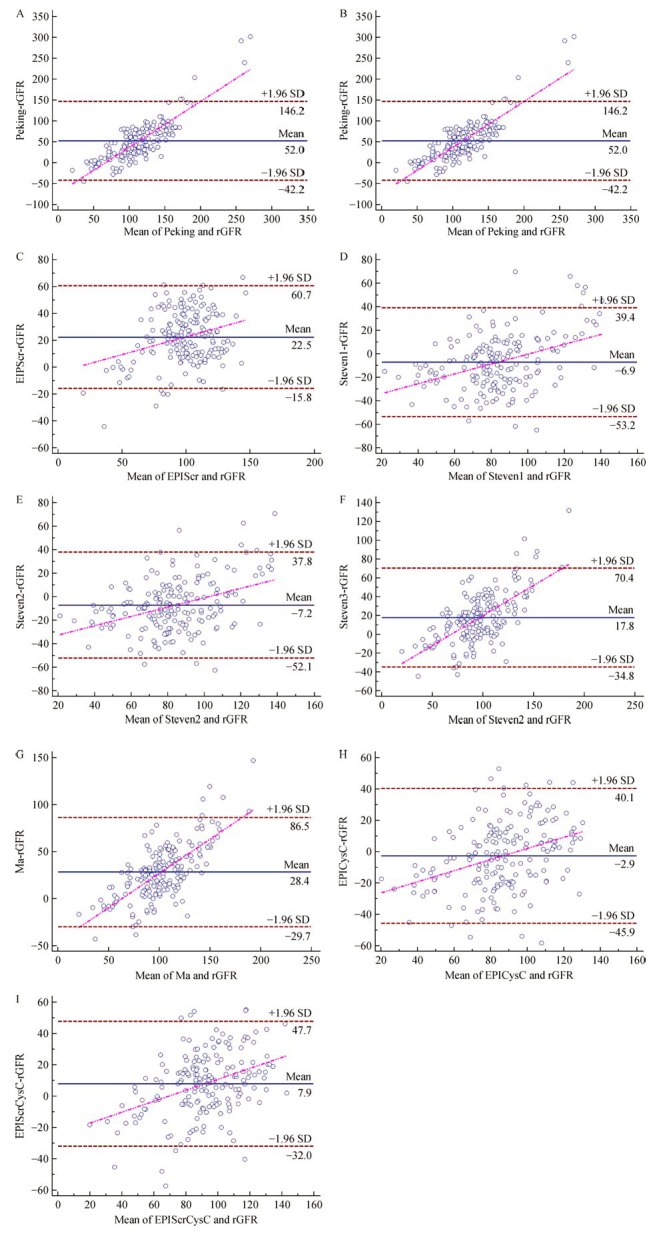
**Bland-Altman analysis of estimated GFR and reference GFR before and after modification.**Horizontal solid line represents the bias of eGFR. Horizontal dashed line represents the 95% confidence interval of standard deviation. Tilting dashed line represents the regression line of bias.

Different equations performed with utterly different accuracies. All the three Scr-based equations overestimated rGFR more than 10 mL/minute/1.73 m^2^. The Peking equation unexpectedly deviated by 16.13 mL/minute/1.73 m^2^. No Scr-based equations had a statisfactory performance, with low P_30_, high IQR and absolute mean bias. The other two kinds of GFR equations predicted relatively accurate estimates. The Scy C-based and Scr-Scy C combination based equations were similarly accurate. Among them, the CKD-EPI_Scr-Scys C_ equation performed the best according to the absolute mean bias and P_30 _( ***Table 3***). Misclassification analysis of CKD stages and Bland-Altman analysis also indicated that the CKD-EPI_Scr-Scys C_ equation performed well ( ***Table 4*** and *** Fig. 1***).

**Tab.3:** Performance of GFR estimation equations in the overall sample

**Equation**	**Mean bias**	**Absolute mean bias**	**IQR**	** P**_**30**_** (%)**
**Scr-based**				
MDRD	31.91^**^	35.61^**^	39.61	47.8
Peking	52.03^**^	54.44^**^	53.22	28.3^**^
CKD-EPI_ Sc__r_	22.52^**^	25.21^**^	27.67	52.8
**Scys C-based**				
Steven 1	-6.83^**^	19.54^**^	30.61	71.1^**^
Steven 2	-7.12^**^	19.36^**^	29.35	72.8^**^
CKD-EPI_Scys C_	-2.86^**^	18.20^**^	30.73	73.3^**^
**Scr and Scys C -based**				
Ma	-2.86^**^	18.20^**^	30.73	73.3^**^
Steven 3	18.87^**^	24.50^**^	33.37	62.8^**^
CKD-EPI_Sc__r__-Scys C_	7.90^**^	17.77^**^	24.84	73.3^**^

Mean Bias: eGFR–rGFR, mL/minute/1.73 m^2^; Absolute Mean Bias:| eGFR–rGFR|, mL/minute/1.73 m^2^; IQR: (75%–45%) limits of agreement of the equations, mL/minute/1.73 m^2^; P_30_: the percentage of eGFR within 30 % of rGFR; ^**^ *P*<0.001, compared with the rGFR.

**Tab.4:** CKD misclassification in the additional external validation data set

**Equation**	**CKD stage**			**Misclassification of CKD stage**
	**Stage 1**	**Stage 2**	**Stage 3–5**	
rGFR	84	74	22	—
**Scr-based**				
MDRD	140(24%)	27(41%)	13(7%)	74(41%)
Peking	152(47%)	16(50%)	12(0)	80(44%)
CKD-EPI_ Sc__r_	153(40%)	16(63%)	11(0)	71(39%)
**Scys C-based**				
Steven 1	54(21%)	91(44%)	35(51%)	71(39%)
Steven 2	57(22%)	89(40%)	34(47%)	63(35%)
CKD-EPI_Scys C_	75(32%)	76(41%)	29(45%)	68(38%)
**Scr and Scys C -based**				
Ma	137(45%)	26(50%)	17(12%)	77(43%)
Steven 3	123(41%)	37(32%)	20(25%)	71(39%)
CKD-EPI_Sc__r__-Scys C_	116(38)	42(28%)	22(31%)	69(38%)

**Note:** Data are presented as number of each CKD stage patients(number of underestimation of CKD stage patients). Misclassification is defined as the proportion of patients with an unequal CKD stage between rGFR and the eGFR. Underestimation of CKD stage=CKDstage_rGFR_ - CKDstage_eGFR_≧ 1.

### Accuracy of the equations in the subgroups

Consistent with the whole population, the Scr-based equations obviously overestimated GFR both in different subgroups of hematopathy and different CKD stages. Additionally, CKD-EPI_Scys C_ equation in the rGFR ≧ 90 mL/minute/1.73 m^2^ subgroup and Steven 2 equation in the rGFR<90 mL/minute/1.73 m^2^ subgroup provided relatively more accurate estimates in each subgroup. CKD-EPI_Scr-Scys C_ predicted the most precise eGFR both in the lymphoma and leukemia subgroups ( ***Table 5–6***).

**Tab.5:** Performance of the nine equations in different types of hematopathy

**Equation**	**Lymphoma**				**Leukemia**			
	**Mean bias**	**Absolute mean** **bias**	**IQR**	**P_30_(%) **	**Mean bias**	**Absolute mean** **bias**	**IQR**	**P_30 _(%) **
**Scr-based**								
MDRD	24.37	28.24	35.08	50.6	47.09	48.12	44.47	38.1^**^
Peking	41.00^**^	43.11^**^	45.38	32.2	72.42	72.81^**^	52.84	49.21^**^
CKD-EPI_ Sc__r_	22.43^**^	25.00^**^	25.09	49.4^**^	-3.72^**^	28.47^**^	30.0	71.4^**^
**Scys C-based**								
Steven 1	-9.29^**^	19.59^**^	29.12	71.3^**^	-2.82^**^	19.76^**^	32.46	73.0^**^
Steven 2	-9.30^**^	19.44^**^	29.73	71.3^**^	-3.47^**^	19.47^**^	32.81	79.4^**^
CKD-EPI_Scys C_	-4.11^**^	19.25^**^	30.9	73.6^**^	16.91^**^	19.85^**^	26.89	71.4^**^
**Scr and Scys C -based**								
Ma	22.19^**^	27.30	30.94	51.7^**^	40.58^**^	41.26^**^	35.53	68.5^**^
Steven 3	12.47^**^	20.36^**^	4.52	70.1^**^	28.87^**^	30.72^**^	33.24	79.2^**^
CKD-EPI_Sc__r__-Scys C_	6.06^**^	17.89^**^	24.94	77^**^	13.91^**^	17.85^**^	24.79	85.7^**^

Mean bias: eGFR–rGFR, mL/minute/1.73 m^2^; Absolute mean bias:|eGFR–rGFR|, mL/minute/1.73 m^2^; IQR: (75%–45%) limits of agreement of the equations, mL/minute/1.73 m^2^; P_30_: the percentage of eGFR within 30 % of rGFR; ^**^ *P*<0.001, compared with the rGFR.

**Tab.6:** Performance of the nine equations in different CKD stages

**Equation**	**rGFR<90 mL/minute/1.73 m**^2^				**rGFR **≥** 90 mL/minute/1.73 m**^2^			
	**Mean bias**	**Absolute mean** **bias**	**IQR**	P_**30 **_(%)	**Mean bias**	**Absolute mean** **bias**	**IQR**	P_**30 **_(%)
**Scr-based**								
MDRD	29.52^**^	33.00	40.00	38.5	34.64^**^	38.59	77.84	58.3
Peking	60.27^**^	47.53^**^	55.77	26.0	16.51^**^	62.34^**^	39.64	31.0
CKD-EPI_ Sc__r_	17.13^**^	29.74^**^	25.94	32.3^**^	14.25^**^	20.03^**^	19.93	76.2^**^
**Scys C-based**								
Steven 1	-9.71^**^	14.79^**^	24.71	72.9^**^	2.73^**^	24.97^**^	39.1	69.0
Steven 2	-9.73^**^	15.21^**^	22.77	75.0^**^	0.84^**^	24.10	35.45	70.2
CKD-EPI_Scys C_	-6.38^**^	16.62^**^	28.81	69.8^**^	0.88^**^	20.00	31.53	77.4
**Scr and Scys C -based**								
Ma	32.59^**^	28.93^**^	30.42	39.6	8.26^**^	37.01^**^	44.93	52.4^**^
Steven 3	20.27^**^	21.59^**^	28.37	60.4^**^	6.59	27.81^**^	38.29	65.5^**^
CKD-EPI_Sc__r__-Scys C_	5.17^**^	16.55^**^	25.41	70.8^**^	4.61^**^	19.17^**^	24.28	76.2^**^

Mean bias: eGFR–rGFR, mL/minute/1.73 m^2^; Absolute mean bias:|eGFR–rGFR|, mL/minute/1.73 m^2^; IQR: (75% - 45%) limits of agreement of the equations, mL/minute/1.73 m^2^; P_30_: the percentage of eGFR within 30 % of rGFR; ^**^ *P*<0.001, compared with the rGFR.

## Discussion

Renal dysfunction is a common side effect of chemotherapeutic agents, and a number of case reports suggested that it may be associated with acute renal failure^[27–32]^. Some reports also suggested that this adverse effect may be caused by two possible mechanisms: tumor lysis syndrome, with precipitation and deposition of uric acid in the renal tubules, and toxic tubular damage. Tubular cells are susceptible to the toxic effects of drugs, as they have a role in concentrating and reabsorbing the glomerular filtrate, what exposes them to high levels of circulating toxins^[33]^. However, the early period of CKD is asymptomatic, which means people do not get identified or treated until the disease has progressed to near end-stage kidney failure. Therefore, a precise, non-invasive and repeatable method is eager for periodically assessing kidney function for hematological patients. According to these facts, both K/DOQI and KDIGO practice guidelines for evaluation and management of CKD^[13]^ recommended that use of GFR estimation equations for assessing kidney function. Furthermore, the lower the GFR level is, the higher the monitor frequencies are^[34–36]^.

Factors affecting the accuracy of GFR evaluation equations have been controversial^[37]^. Up to now, the recognized main influences on the accuracy of equations include design of the study, ethnicity, kidney function parameter, sample size and GFR level^[37]^. Whether the primary disease of CKD affects the accuracy of equations or not is uncertain. Or rather, whether one or a few "representative" equations could predict similar accuracy for different CKD patients is not able to draw an absolute conclusion. Thus, studies worldwide successively validated equations in various patients population to learn their accuracy for various target populations^[34,38–39]^.

A meta-analysis indicated that the CKD-EPI_Scr-Scys C_ equation was more accurate than the MDRD equation in categorizing the risk of mortality and CKD progression to ESRD^[40]^. Another recent systematic review in hematological recipients study demonstrated that CKD-EPI_Scr-Scys C_ equation was superior to other included equations^[41]^. The results of this study found that the Scr-based equation obviously overestimated GFR both in different subgroups of hematopathy and different CKD stages. On the other hand, Scy C-based equations provided relatively more accurate estimates, CKD-EPI_Scr-Scys C_ predicting the most precise eGFR. These results were similar to those of the previous two meta-analyses, showing a hypothesis that the accuracy of the equations might be irrelevant with the primary disease of CKD, but closely with the design of the study, kidney function parameter and GFR level. The CKD-EPI_Scr-Scys C_ equation would be generally suitable for hematological patients, regardless of the type of diseases.

Some study used the inulin single-injection method as the GFR reference standard. This study set the ^99m^Tc-DTPA kidney dynamic imaging as the GFR reference standard, which has been proved inferior to inulin clearance^[42]^. The principal limitations of the kidney dynamic imaging consist in clinical experiences and region of interest sketching by operators, which is slightly subjective. However, once the operators are experienced, the kidney dynamic imaging could also obtain an ideal performance, such as this study. Additionally, we consistently applied Gates method as the reference standard, not only in our modification studies but in new equation development studies^[43–48]^. Consequently, we always put the quality of Gates method at the first step. We examined the accuracy of GFR from the Gates method time and again. Of course, to dismiss the puzzle, our group have gradually developed dynamic dual plasma method and worked harder to get more accurate data.

In conclusion, the accuracy of the GFR equations in this study did not achieve a satisfactory accuracy in hematological patients. Therefore, it is imminent to modify some equations or develop a new GFR equation for this sample. In this study, CKD-EPI_Scr-Scys C_ equation was suitable for renal function screening in whole patients of hematopathy. CKD-EPI_Scys C_ equation in the rGFR ≧ 90 mL/minute/1.73 m^2^ subgroup and the Steven 2 equation in rGFR<90 mL/minute/1.73 m^2^ subgroup could be recommended for monitoring kidney function in each subgroup.
